# A multimodal analysis suggests partial IRF8/TLR7-associated myeloid transcriptional convergence between diabetic kidney disease and Parkinson’s disease

**DOI:** 10.1371/journal.pone.0356691

**Published:** 2026-08-20

**Authors:** Xue Hu, Yingzhuo Li, Bosheng Luo, Yang Wang, Chunjuan Xia, Jiaping Wang

**Affiliations:** 1 Department of Radiology, Second Affiliated Hospital of Kunming Medical University, Kunming, Yunnan, China; 2 Department of Ultrasound, Second Affiliated Hospital of Kunming Medical University, Kunming, Yunnan, China; Augusta University, TAIWAN

## Abstract

**Background:**

Diabetic kidney disease (DKD) and Parkinson’s disease (PD) affect different organs but share epidemiological associations and innate immune abnormalities. The extent and cellular basis of reproducible cross-disease transcriptomic convergence remain uncertain.

**Methods:**

Two DKD glomerular and three PD substantia nigra microarray cohorts were analyzed independently and combined using disease-specific and cross-disease meta-analysis. Independent DKD single-cell RNA-sequencing, PD single-nucleus RNA-sequencing, and spatial transcriptomic datasets were used for cell-type localization and donor- or sample-level analyses. CellChat, Monocle2, scTenifoldKnk, and previously generated UNAGI-compatible outputs were used to evaluate predicted intercellular communication, transcriptional-state continua, in-silico IRF8 perturbation, and compound prioritization. Threshold, leave-one-cohort-out, root-orientation, downsampling, and statistical-unit sensitivity analyses were performed where applicable.

**Results:**

The original nominal-threshold intersection of 36 genes was not retained after cohort-wise multiple-testing correction. Genome-wide overlap between DKD and PD was limited in the expanded analysis. IRF8 and TLR7 showed positive effect directions in both diseases but did not pass cross-disease false-discovery-rate correction and were therefore treated as exploratory candidates. Donor-level analyses did not detect statistically significant increases in broad myeloid-lineage proportions, although the confidence intervals did not establish equivalence. Selected DKD CCL and TNF interactions were retained in both of two targeted balanced-downsampling iterations, whereas the PD TGF-β signal was sampling-sensitive. Monocle2 identified branched and partially overlapping transcriptional-state continua. Spatial analyses provided marker-supported localization evidence without treating individual spatial observations as independent replicates. In-silico IRF8 deletion predicted regulatory-network changes but was not experimentally validated. NVP-AUY922 was the highest-ranked cross-disease computational candidate, but its association was not significant after correction across the matched compound universe.

**Conclusions:**

DKD and PD show limited genome-wide overlap but partial convergence of myeloid- and microglia-enriched inflammatory signals associated with IRF8 and TLR7. These findings provide testable hypotheses rather than evidence of a conserved causal pathway or validated cross-disease treatment.

## 1. Introduction

Diabetic kidney disease (DKD) and Parkinson's disease (PD) represent two of the most burdensome chronic diseases globally, each posing substantial challenges to patients and healthcare systems alike. DKD develops in approximately 40% of patients with type 2 diabetes mellitus and is the leading cause of end-stage renal disease worldwide [1,2]. Its pathophysiology encompasses intraglomerular hypertension, chronic hyperglycemia-driven metabolic perturbations, oxidative stress, and sustained activation of pro-fibrotic and pro-inflammatory signaling cascades, collectively resulting in progressive glomerulosclerosis, tubular atrophy, and interstitial fibrosis [3]. Despite the clinical introduction of renin-angiotensin system blockade, sodium-glucose cotransporter-2 inhibitors, and mineralocorticoid receptor antagonists, a substantial proportion of patients continue to progress toward dialysis dependence, reflecting the incomplete understanding of the disease's core pathological drivers [4].

PD is the second most common neurodegenerative disorder, affecting over 10 million individuals worldwide with incidence rising steeply with age [5,6]. Its defining pathological features include the selective loss of dopaminergic neurons in the substantia nigra pars compacta and the intraneuronal accumulation of misfolded alpha-synuclein into Lewy bodies [7]. Motor manifestations including bradykinesia, rigidity, and resting tremor are accompanied by non-motor symptoms such as cognitive impairment, autonomic dysfunction, and depression. Current therapies, including dopamine replacement and deep brain stimulation, address symptoms but do not modify disease progression, leaving an unmet need for mechanistically grounded therapeutic targets.

Although DKD and PD affect distinct organ systems, epidemiological evidence points to a meaningful comorbidity between them. A meta-analysis of 15 cohort studies including nearly 30 million participants demonstrated that patients with type 2 diabetes carry a significantly elevated risk of developing PD [8]. Individuals with PD also show higher rates of metabolic dysregulation and renal impairment relative to age-matched controls [9]. These observations raise a biologically important question: whether the two diseases share common pathogenic mechanisms that operate independently of their organ-specific contexts. Identifying such shared mechanisms could have direct clinical relevance, particularly given the aging global population in which both diseases are simultaneously increasing in prevalence.

A growing body of evidence implicates innate immune dysregulation as a converging pathological theme. In the diabetic kidney, resident macrophages transition from homeostatic to persistently pro-inflammatory states under metabolic stress, driving tubular injury and fibrotic remodeling through TLR-mediated and NF-kB-dependent signaling [10,11]. In the Parkinson's brain, chronic microglial activation perpetuates dopaminergic neuronal loss through sustained cytokine release and reactive oxygen species production [12]. In both settings, resident myeloid cells undergo intrinsic transcriptional reprogramming toward a hyperinflammatory phenotype that amplifies and sustains organ damage beyond the scope of the initiating insult. This cellular parallel suggests that the comorbidity of DKD and PD may be rooted in a conserved myeloid inflammatory program rather than coincidental overlap of independent pathological processes.

Key candidates for mediating such a program include IRF8 and TLR7. IRF8 is a transcription factor required for the development and maturation of myeloid cells, and governs downstream inflammatory effectors including NF-kB and HIF1A signaling components [13]. In microglia specifically, IRF8 defines the epigenetic landscape and directs cell-type-specific transcriptional programs; its dysregulation has been identified as a critical driver of reactive microglial states in neurological injury models [14]. TLR7 is a pattern recognition receptor activated by single-stranded RNA and endogenous damage-associated molecular patterns, serving as an upstream driver of innate immune activation in both macrophages and microglia [15]. These two molecules may collectively underpin a shared transcriptional state induced by deteriorating tissue microenvironments characterized by extracellular matrix disorganization, aberrant chemokine signaling, and sterile ligand accumulation. However, whether IRF8 and TLR7 co-activation constitutes a genuine cross-organ shared mechanism in human DKD and PD at single-cell resolution remains to be rigorously established.

The development of single-cell RNA sequencing, spatial transcriptomics, pseudotime trajectory inference, and AI-driven perturbation modeling now provides the tools necessary to address these questions systematically. These approaches enable cell-type-resolved dissection of disease transcriptomes, reconstruction of cell state dynamics over disease progression, spatial mapping of gene expression to defined tissue niches, and in silico evaluation of therapeutic candidates. Applied within a comparative cross-disease framework, they offer a means to determine whether the pathological convergence of DKD and PD reflects a shared molecular architecture at the level of individual cell populations.

In the present study, we used an integrative, hypothesis-generating framework to evaluate the extent of transcriptomic concordance between DKD and PD. We first expanded the bulk analysis to five independently processed microarray cohorts and performed disease-specific and cross-disease meta-analyses. We then evaluated the cell-type distribution of exploratory candidates, donor-level myeloid-lineage proportions, condition-specific intercellular communication, transcriptional-state continua, spatial localization, predicted regulatory consequences of IRF8 perturbation, and cross-disease compound rankings. Our objective was to identify reproducible points of convergence while clearly distinguishing statistically supported findings from exploratory computational predictions. The analyses were not designed to establish that DKD and PD share an identical causal mechanism or that the prioritized genes and compounds represent validated therapeutic targets.

## 2. Materials and methods

### 2.1 Data acquisition and study design

Publicly available transcriptomic datasets were retrieved from the Gene Expression Omnibus database [16]. The expanded bulk transcriptomic analysis included two DKD glomerular microarray cohorts and three PD substantia nigra microarray cohorts. GSE30528 included 9 DKD and 13 control samples, GSE96804 included 41 DKD and 20 control samples, GSE7621 included 16 PD and 9 control samples, GSE20292 included 11 PD and 15 control samples, and GSE49036 included 8 late-stage PD and 8 stage-0 control samples. Complete information regarding tissue source, platform, disease definition, available clinical covariates, inclusion criteria, and analytical use is provided in S1A–S1C Tables.

The DKD single-cell RNA-sequencing dataset was obtained from GSE209781 and included three DKD and three control kidney donors. The PD single-nucleus RNA-sequencing dataset was obtained from GSE184950. Multiple samples originating from the same PD donor were linked using donor identifiers before donor-level statistical analysis. Spatial transcriptomic analyses used the processed DKD dataset GSE211785 and the PD spatial dataset GSE253975. Kidney and brain datasets were not directly merged across diseases. Each dataset was processed within its original tissue and platform context, and cross-disease interpretation was based on concordance of independently estimated effects and cell-state patterns.

### 2.2 Cohort-specific differential expression and meta-analysis

Each microarray cohort was processed and analyzed independently to avoid direct integration of expression measurements generated using different platforms. Expression values were log2-transformed when required. Platform-specific annotation files were used to map probes or transcript clusters to gene symbols, and multiple probes mapping to the same gene were averaged before differential-expression analysis.

Disease-versus-control comparisons were performed using limma with empirical-Bayes moderation. Available covariates were included only when they were sufficiently complete and variable within the corresponding cohort. Age and sex were available for GSE20292, sex was available for GSE96804, and RNA-integrity measurements were available for GSE49036. Post-mortem interval, disease severity, and other clinical factors could not be adjusted uniformly because they were unavailable or incomplete in several public datasets. Benjamini–Hochberg correction was applied within each cohort [17].

Gene-level log2 fold changes and standard errors were carried forward to disease-specific inverse-variance meta-analysis. The two DKD cohorts were combined using a fixed-effect model, whereas the three PD cohorts were combined using a random-effects model estimated by restricted maximum likelihood. An all-cohort random-effects model and a disease-category moderator analysis were additionally used to characterize overall effects and between-disease heterogeneity. Multiple-testing correction was performed across the 12,382 genes measured in all five cohorts.

The primary definition of a meta-supported cross-disease gene required concordant effect directions and FDR < 0.05 in the DKD, PD, and all-cohort analyses. Genes showing concordant directions without satisfying all primary criteria were retained only as exploratory candidates. Sensitivity analyses evaluated FDR thresholds of 0.05 and 0.01 together with minimum absolute disease-level meta-effect thresholds of 0, 0.2, and 0.5. Leave-one-cohort-out analyses were performed by excluding each cohort in turn and recalculating the corresponding effects. Complete gene-level statistics, sensitivity analyses, and leave-one-cohort-out results are provided in S1D–S1K Tables.

Functional enrichment analyses were performed for the exploratory candidate sets using Gene Ontology and Kyoto Encyclopedia of Genes and Genomes annotations. The enrichment background comprised genes retained and tested in the relevant differential-expression analysis. Because the candidate set was sensitive to statistical thresholds, enrichment results were interpreted as hypothesis-generating rather than as evidence of a universally conserved pathway [18].

### 2.3 Single-cell and single-nucleus processing

The DKD single-cell and PD single-nucleus datasets were processed using Seurat. Cells or nuclei with fewer than 200 detected genes or mitochondrial transcript fractions greater than 20% were excluded [19]. Data were normalized and scaled, principal-component analysis was performed, and sample-associated technical variation was reduced using Harmony [20]. UMAP was used for visualization and clustering. Major lineages were annotated using canonical markers, and renal myeloid cells and brain microglial/myeloid cells were subsequently extracted for focused analyses and high-resolution subclustering.

The comparative single-cell analysis was used to evaluate the cellular distribution of exploratory genes nominated by the bulk meta-analysis. Expression localization in UMAP, dot plots, feature plots, and violin plots was interpreted descriptively and was not treated as independent donor-level differential-expression evidence.

### 2.4 Donor-level cell-composition analysis

Cell-type proportions were calculated using the donor, rather than the individual cell, as the biological replicate. For DKD, each sample represented one donor. For PD, cells from multiple samples originating from the same donor were aggregated before calculation of donor-level proportions. The broad myeloid-lineage proportion was calculated as the number of cells assigned to the relevant myeloid or microglial/myeloid category divided by the total number of quality-controlled cells from the same donor.

Group differences were evaluated using two-sided Wilcoxon rank-sum tests. In addition to P values, we calculated the Hodges–Lehmann disease-minus-control location shift and its 95% confidence interval. Cliff’s delta was used as a distribution-free effect-size measure, with confidence intervals estimated using 10,000 stratified bootstrap resamples.

Because one PD control donor contained only 80 retained cells, sensitivity analyses were performed using minimum thresholds of 100, 500, and 1,000 retained cells per donor. Non-significant P values were not interpreted as evidence of equivalent cell-type proportions. Donor-level source data, descriptive statistics, effect sizes, and sensitivity analyses are provided in S2A–S2E Tables.

### 2.5 Cell–cell communication analysis

Intercellular communication was inferred using CellChat v1.6.1 and CellChatDB.human. Four condition-specific objects were analyzed separately: DKD control, DKD disease, PD control, and PD disease [21]. Communication probabilities were calculated using the trimean expression estimator with raw.use = TRUE. The primary analysis used population.size = TRUE, 100 bootstrap permutations, and a minimum of 10 cells per cell population. Interactions with empirical P < 0.05 were retained. Empirical P values returned as zero under 100 permutations were reported as P < 0.01.

Complete ligand–receptor results were exported with the sending population, receiving population, ligand, receptor or receptor complex, signaling pathway, communication probability, empirical P value, disease, and condition. TGF-β, TNF, and CCL interactions were additionally extracted as prespecified pathways of interest.

To assess sensitivity to cell-number imbalance and random cell selection, two independent balanced downsampling analyses were conducted. Shared cell types were capped at 300 cells per condition; PD lymphocytes were capped at 225 cells because only 225 control lymphocytes were available. Control and disease groups were analyzed separately using the same inference and filtering parameters, with population.size = FALSE after balancing. A completed iteration with no significant target-pathway interaction was counted as a zero-interaction result rather than as a failed run. Recurrence frequencies and disease–control differences are reported in S4D–S4F Tables. Because only two resampling replicates were performed, this analysis was interpreted as a targeted sensitivity assessment rather than an exhaustive robustness analysis.

### 2.6 Pseudotime and root-state sensitivity analysis

Monocle2 was used to reconstruct descriptive transcriptional-state continua within DKD myeloid cells and PD microglial/myeloid cells [22]. Trajectories were constructed using 400 data-driven ordering genes selected according to empirical dispersion. The mean-expression–empirical-dispersion relationships and ordering-gene selection diagnostics for DKD and PD are shown in S3A–S3B Fig. Candidate genes evaluated downstream were not specifically selected to construct the trajectory.

Dimensionality reduction was performed using DDRTree. Terminal nodes were defined as principal-graph nodes with degree 1, and branch nodes were defined as nodes with degree greater than 2. The primary root was selected from a control-enriched terminal State with adequate cell number and donor coverage. A disease-enriched terminal State located at the opposite end of the principal graph was used for root-orientation sensitivity analysis.

For DKD, State 5 was selected as the control-enriched primary root and State 1 was used as the reverse-root sensitivity State. For PD, State 4 was selected as the primary root and State 1 was used as the reverse-root State. Complete ordering-gene lists, root-selection criteria, principal-graph vertices and edges, branch information, cell-level trajectory data, and technical diagnostics are provided in S3A–S3H Tables.

Because the input datasets were cross-sectional, pseudotime was interpreted as a computational ordering of heterogeneous transcriptional states rather than direct longitudinal disease progression. Correlations between pseudotime and quality-control metrics were included in the diagnostic assessment, and reverse-root results were used to evaluate sensitivity to trajectory orientation.

### 2.7 Spatial transcriptomic analysis

The processed DKD spatial object contained 37,143 matched spatial observations. The “Mac/Mono” label was inherited from the processed GSE211785 annotation rather than generated through a new reference-based deconvolution procedure [16]. Marker support was assessed using available members of a macrophage and monocyte panel, including LST1, TYROBP, FCER1G, CTSS, AIF1, LYZ, CD68, C1QA, C1QB, C1QC, S100A8, and S100A9. IRF8, TLR7, MS4A6A, SAMHD1, and CPVL were evaluated after annotation and were not used to define the Mac/Mono population.

The PD spatial analysis included 10 sample files, comprising five control and five PD samples, with 11,859 spatial observations in total. Spatial clustering was performed using Seurat after normalization and variable-feature selection. Principal-component analysis was followed by clustering using the first 15 principal components where supported and a resolution of 0.5. Cluster identities were interpreted using TH and SLC6A3 for dopaminergic neurons, AIF1 and CX3CR1 for microglial/myeloid signals, GFAP and AQP4 for astrocytes, MBP and PLP1 for oligodendrocytes, CD8A and CD3E for T-cell-associated signals, and VWF and PECAM1 for vascular endothelial cells. The cluster with the highest combined vascular and immune marker support was descriptively labeled “Vascular/Immune.” This label represents a marker-supported interpretation of an unsupervised cluster rather than a histologically validated anatomical compartment.

No additional reference-based spatial deconvolution was performed. Observation-level quality-control summaries included total counts, detected genes, zero-library observations, and mitochondrial read fraction where available. To avoid spatial pseudoreplication, gene-expression values were first summarized independently within each biological sample or section. Group comparisons in Fig 5B were then conducted using these sample- or section-level summaries rather than pooled individual spots. Dataset structure, observation counts, quality-control results, annotation criteria, and statistical-unit audits are provided in S5A–S5F Tables.

### 2.8 In-silico IRF8 perturbation

The predicted regulatory consequences of IRF8 loss were evaluated using the scTenifoldKnk R package. This analysis was conducted independently of the UNAGI and iDREM analyses [23]. Raw gene-by-cell count matrices from unperturbed control myeloid or microglial populations were used to reconstruct baseline gene-regulatory networks.

The virtual knockout was specified using gKO = “IRF8.” In scTenifoldKnk, the pseudo-knockout network is generated by removing the inferred outgoing regulatory connections of the target gene from a copy of the baseline network. The baseline and pseudo-knockout networks were compared using manifold alignment, and genes were ranked according to predicted differential regulation.

The explicitly specified parameters were qc = FALSE, nc_nNet = 10, nc_nCells = 500, and nc_nComp = 3; remaining arguments were retained at the installed package defaults. Because qc = FALSE, qc_mtThreshold = 0.1 and qc_minLSize = 1000 were not applied. Genes with |Z| > 2 and Benjamini–Hochberg-adjusted P < 0.05 were highlighted.

The resulting values represent predicted differences between inferred baseline and pseudo-knockout regulatory networks. They do not represent experimentally measured expression changes after IRF8 deletion. The disease-specific predictions were not validated using matched IRF8-knockout, CRISPR, or Perturb-seq data.

### 2.9 UNAGI-based compound prioritization

Existing disease-specific compound rankings generated by the UNAGI-compatible perturbation workflow were reused without retraining the UNAGI models [23]. Scores were oriented such that higher values represented stronger predicted opposition to the disease-associated transcriptional state.

Because the raw DKD and PD scores were not necessarily measured on identical scales, they were independently converted to within-disease mid-rank percentiles: p = (r − 0.5)/N, where r denotes the rank of the compound or compound group and N denotes the number of ranked entries in the corresponding disease. For compounds represented by multiple source rows, the median oriented score was used in the compound-level analysis.

Compounds represented in both disease rankings were assigned a dual-disease score calculated as the harmonic mean of the two percentiles: Sdual = 2pDKDpPD/(pDKD + pPD). This formulation prioritized entries that ranked consistently highly in both diseases and penalized candidates with a high rank in only one disease. Cross-disease empirical significance was evaluated by permuting PD compound labels relative to the fixed DKD labels 10,000 times. One-sided empirical P values were calculated and adjusted across the matched compound universe using the Benjamini–Hochberg procedure.

The UNAGI-compatible resource retained compound-to-gene regulatory relationships, adjusted statistical values, and iDREM-suggested regulatory directions where available. Individual CMAP perturbation identifiers, cell lines, treatment doses, and treatment durations were not retained and could not be reconstructed from the existing output [24]. Complete rankings and separate top-30 and bottom-30 results are provided in S6A–S6F Tables.

## 3. Results

### 3.1 Expanded microarray meta-analysis reveals limited genome-wide overlap but concordant IRF8/TLR7 trends

**Fig 1:**
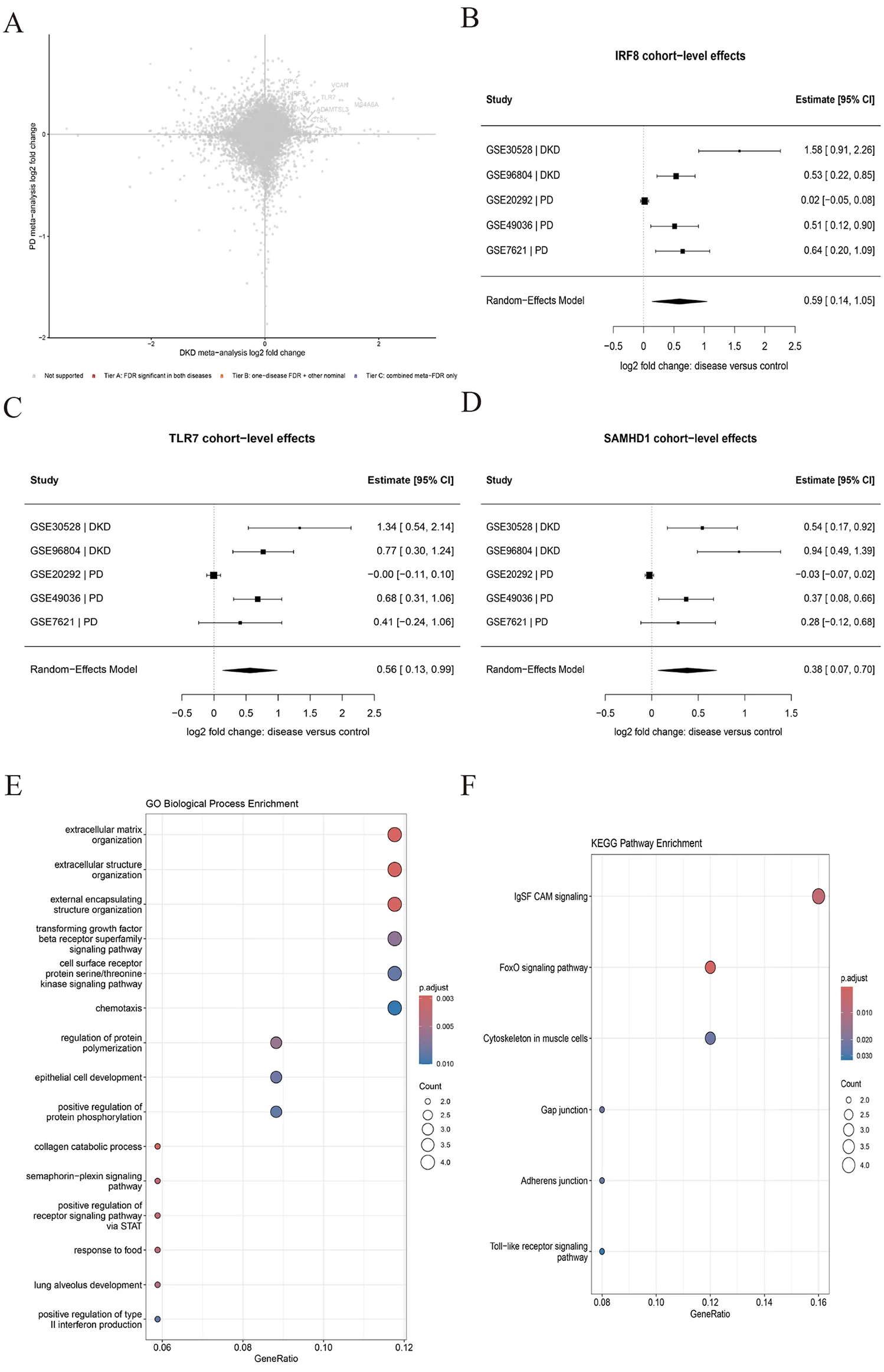
Expanded cross-disease microarray meta-analysis. (A) Comparison of DKD and PD disease-level meta-effect estimates across genes measured in all five cohorts. (B–D) Cohort-specific and pooled effect estimates for IRF8, TLR7, and SAMHD1, respectively. Points represent estimated log2 fold changes and horizontal lines indicate confidence intervals. (E) Gene Ontology enrichment of the exploratory directionally concordant candidate set. (F) KEGG enrichment of the same candidate set. Enrichment analyses are hypothesis-generating because the candidate set was sensitive to statistical and effect-size thresholds.

We expanded the bulk transcriptomic analysis to two DKD glomerular cohorts and three PD substantia nigra cohorts. Each cohort was analyzed independently, followed by disease-specific meta-analysis and cross-disease comparison of the resulting gene-level effects (Fig 1A–1D). This approach avoided direct merging of expression values produced using different microarray platforms.

The original nominal-threshold intersection nominated 36 candidate genes. However, this set was not retained after cohort-wise BH correction and was therefore no longer interpreted as a robust shared DEG signature. Across the 12,382 genes measured in all five cohorts, six genes reached FDR < 0.05 in both disease-specific analyses when no minimum meta-effect threshold was imposed. None remained when minimum absolute disease-level meta-effects of 0.2 or 0.5 were required, indicating that the estimated genome-wide overlap was limited and sensitive to the effect-size criterion (S1D-S1F Fig; S1E–S1H Tables).

None of the 12 originally prioritized upregulated genes passed genome-wide FDR correction in the PD or combined cross-disease analysis. IRF8 showed a positive DKD meta-effect of log2FC = 0.722 with FDR = 5.24 × 10^-6^ and a positive but non-significant PD meta-effect of log2FC = 0.342 with P = 0.0985 and FDR = 0.672. Its combined effect was log2FC = 0.593 with nominal P = 0.0106 and FDR = 0.344. TLR7 similarly showed a positive DKD meta-effect of log2FC = 0.915 with FDR = 5.84 × 10^-5^ and a positive but non-significant PD effect of log2FC = 0.324 with P = 0.164 and FDR = 0.672. IRF8 and TLR7 retained concordant positive effect directions in leave-one-cohort-out analyses but did not satisfy the criteria for genome-wide significant shared DEGs (S1E Fig; S1I-S1K Tables).

IRF8, TLR7, MS4A6A, SAMHD1, and CPVL were therefore carried forward as exploratory, directionally concordant candidates for cell-type-resolved evaluation. GO and KEGG analyses of the exploratory candidate sets highlighted extracellular-matrix organization, chemotaxis, innate immune signaling, and cell-communication terms (Fig 1E-1F). Because the candidate lists were threshold-sensitive, these enrichment results were interpreted as hypothesis-generating.

### 3.2 Comparative single-cell atlases identify the principal renal and brain cell populations

**Fig 2:**
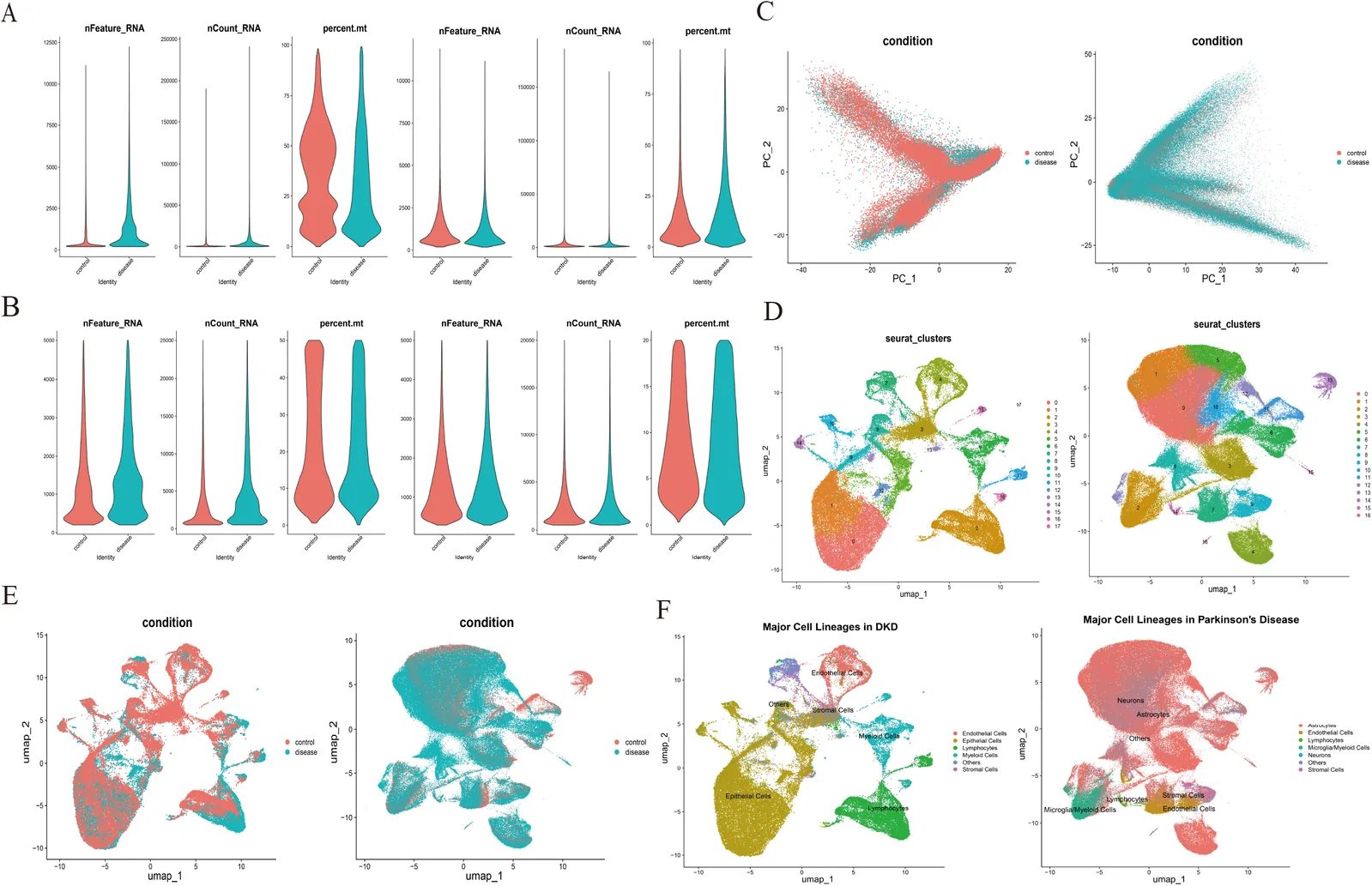
Construction of comparative DKD single-cell and PD single-nucleus atlases. (A) Quality-control distributions before filtering in the DKD and PD datasets. (B) Quality-control distributions after filtering. (C) PCA visualization by disease condition. (D) UMAP visualization of unsupervised clusters. (E) UMAP visualization by disease condition. (F) UMAP visualization of annotated major cell populations. DKD and PD datasets were processed independently and were not merged across organs.

After quality control, normalization, and Harmony correction, PCA and UMAP were used to visualize the DKD kidney and PD substantia nigra datasets (Fig 2A–2E). Cells and nuclei were distributed across multiple samples and conditions, although the embeddings were used primarily for visualization and were not considered proof that all biological and technical batch effects had been eliminated.

Canonical markers supported annotation of epithelial, endothelial, stromal, lymphoid, neuronal, glial, renal myeloid, and brain microglial/myeloid populations (Fig 2F). These independently constructed atlases provided the basis for evaluating whether exploratory cross-disease candidates converged preferentially within analogous innate immune populations.

### 3.3 Candidate inflammatory genes are enriched in myeloid populations without a statistically detectable donor-level expansion

**Fig 3:**
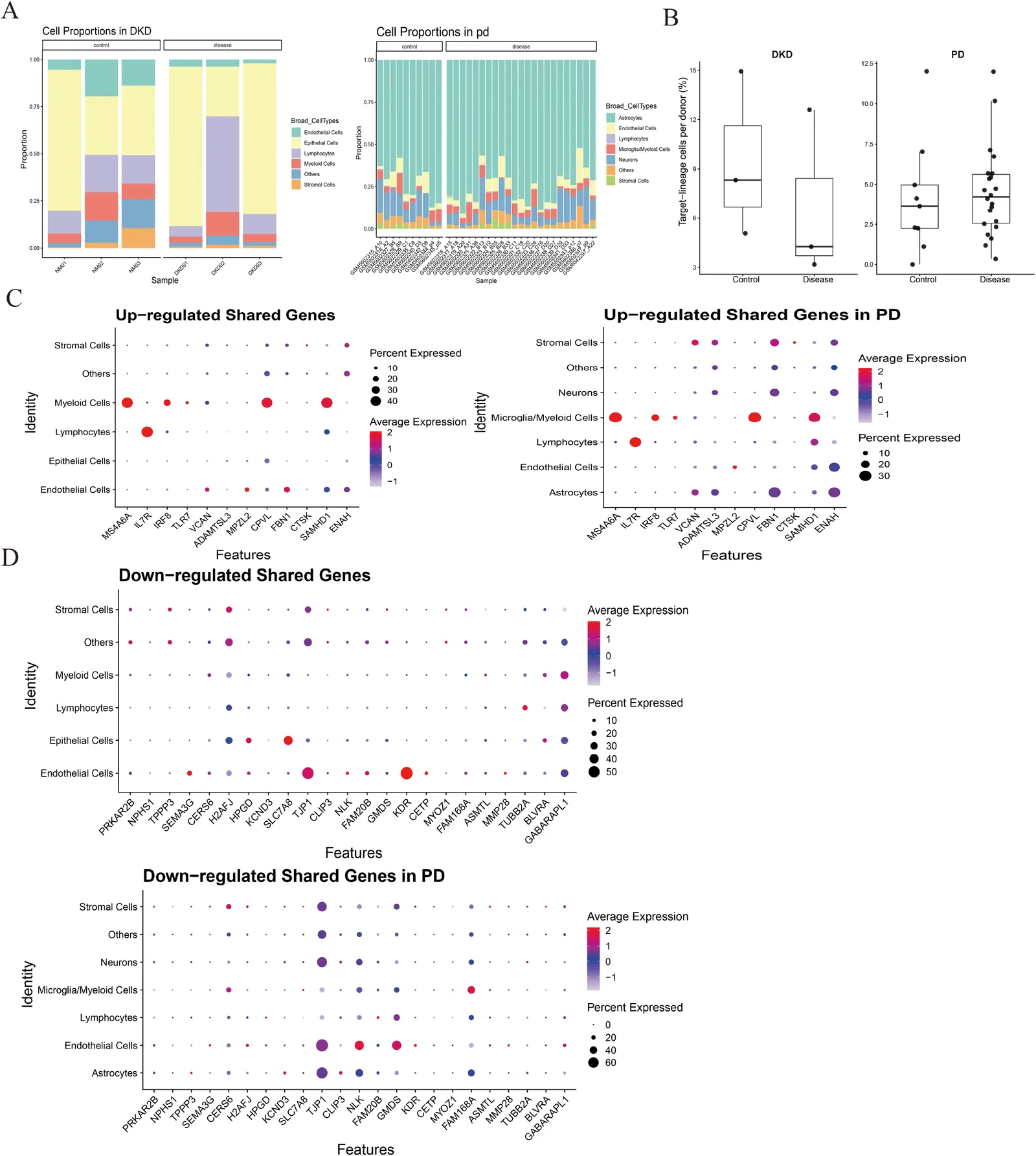
Donor-level myeloid-cell composition and cellular localization of exploratory candidate genes. (A) Major cell-type composition across DKD and PD samples. (B) Donor-level broad myeloid or microglial/myeloid proportions. Each point represents one biological donor. Group comparisons used two-sided Wilcoxon tests, with Hodges–Lehmann shifts and Cliff’s delta reported in S2A–S2D Tables. (C) Dot plots showing the cellular distribution of exploratory positively directed candidates in DKD and PD. (D) Cellular distribution of exploratory negatively directed candidates. Cell-level expression plots are descriptive and do not represent independent donor-level tests.

Cell-type composition differed among individual donors (Fig 3A). Donor-level analysis included three DKD and three control donors, together with 22 PD and nine control donors. Multiple samples from the same PD donor were aggregated before proportion calculation.

In DKD, the median broad myeloid proportion was 4.27% in disease donors and 8.33% in controls. The Hodges–Lehmann disease-minus-control shift was −2.34 percentage points (95% CI, −11.75 to 7.52; P = 0.275), and Cliff’s delta was −0.56 with a bootstrap 95% CI of −1.00 to 0.33. In PD, the median microglial/myeloid proportion was 4.22% in disease donors and 3.65% in controls, corresponding to a shift of 0.50 percentage points (95% CI, −1.68 to 2.99; P = 0.514) and a Cliff’s delta of 0.15 with a bootstrap 95% CI of −0.35 to 0.63 (Fig 3B; S2A-S2D Tables).

Excluding low-cell-count donors did not materially alter the PD result. At a minimum threshold of 100 cells per donor, the estimated shift was 0.13 percentage points (95% CI, −2.28 to 2.24; P = 0.851), and at a 500-cell threshold the shift was 0.08 percentage points (95% CI, −2.29 to 2.17; P = 0.919; S2A Fig and S2E Table).

These analyses did not detect a statistically significant donor-level increase in broad myeloid-lineage abundance. However, the confidence intervals did not establish equivalent proportions, and a quantitative contribution from lineage-abundance changes cannot be excluded, particularly in the small DKD cohort.

IRF8, TLR7, MS4A6A, SAMHD1, and CPVL showed preferential expression within renal myeloid and brain microglial/myeloid populations in the cell-level visualizations (Fig 3C). In contrast, several negatively directed exploratory genes were more prominent in organ-specific parenchymal populations (Fig 3D). These findings support convergence at the level of inflammatory myeloid cell states but do not demonstrate that the same causal program operates identically in both diseases.

### 3.4 Condition-specific CellChat analyses and Monocle2 trajectories reveal pathway- and root-sensitive patterns

**Fig 4:**
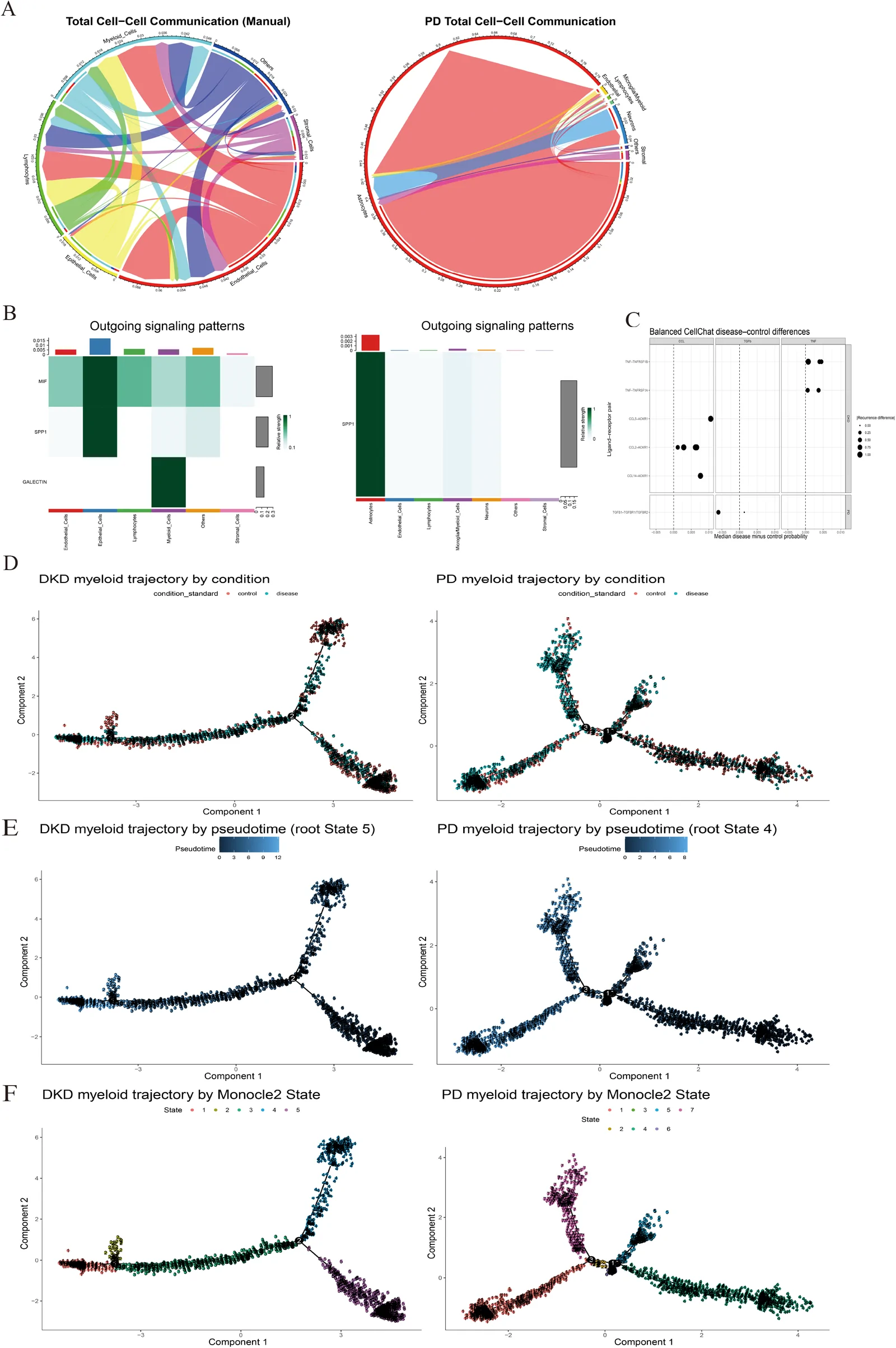
Predicted intercellular communication and Monocle2 transcriptional-state continua. (A) Global CellChat communication networks for DKD and PD. (B) Outgoing signaling-role heatmaps. (C) Disease-minus-control differences in target-pathway communication after condition-specific analysis. (D) DKD and PD trajectories colored by disease condition. (E) The same trajectories colored by pseudotime. (F) The trajectories colored by Monocle2 State. CellChat results represent predicted communication based on ligand–receptor expression. Monocle2 pseudotime represents a computational state ordering and should not be interpreted as directly observed temporal disease progression.

Condition-specific CellChat objects showed extensive predicted communication among parenchymal, stromal, vascular, lymphoid, and myeloid populations (Fig 4A-4B). Disease–control comparisons were performed separately for DKD and PD rather than by pooling the two conditions (Fig 4C).

The DKD disease CCL network showed the greatest reproducibility after balanced downsampling. CCL5–ACKR1, CCL14–ACKR1, and several CCL2–ACKR1 interactions directed toward endothelial cells were retained in both independent resampling replicates. A myeloid-derived CCL2–ACKR1 interaction was retained in one of two replicates and was therefore considered sensitive to random cell selection.

TNF signaling was also detected more reproducibly in DKD disease samples than in controls. Several lymphocyte-derived TNF–TNFRSF1A and TNF–TNFRSF1B interactions were retained in both DKD disease replicates, whereas DKD-control TNF signaling was detected in only one replicate. In contrast, PD TGFB1–TGFBR1/TGFBR2 interactions were retained in only one of two balanced replicates and were interpreted as sampling-sensitive. TGF-β signaling in DKD and CCL or TNF signaling in PD were not detected in either replicate. Complete ligand–receptor and recurrence results are provided in S4A–S4F Tables and S4A Fig.

Monocle2 identified branched transcriptional-state continua in both diseases (Fig 4D–4F). The DKD trajectory comprised five States, four terminal nodes, and two branch nodes. State 5 was selected as a control-enriched primary root, whereas the graph-opposite DKD-enriched State 1 was used for sensitivity analysis. Primary- and reverse-root pseudotime values had a Spearman correlation of −0.792.

The corrected PD trajectory comprised seven States, five terminal nodes, and three branch nodes. State 4 was selected as the control-enriched primary root, and State 1 was used as the graph-opposite PD-enriched sensitivity root. The corresponding primary- and reverse-root pseudotime values had a Spearman correlation of −0.833.

Control and disease cells substantially overlapped across both principal graphs, although their distributions were not uniform. Reverse-root results indicated moderate stability of the overall ordering but confirmed that pseudotime orientation depended on root specification (S3C–S3F Fig). We therefore interpreted the trajectories as descriptive transcriptional-state continua rather than direct temporal reconstruction or perfectly mirrored molecular evolution.

### 3.5 Spatial analyses provide marker-supported localization, and IRF8 perturbation predicts regulatory-network changes

**Fig 5:**
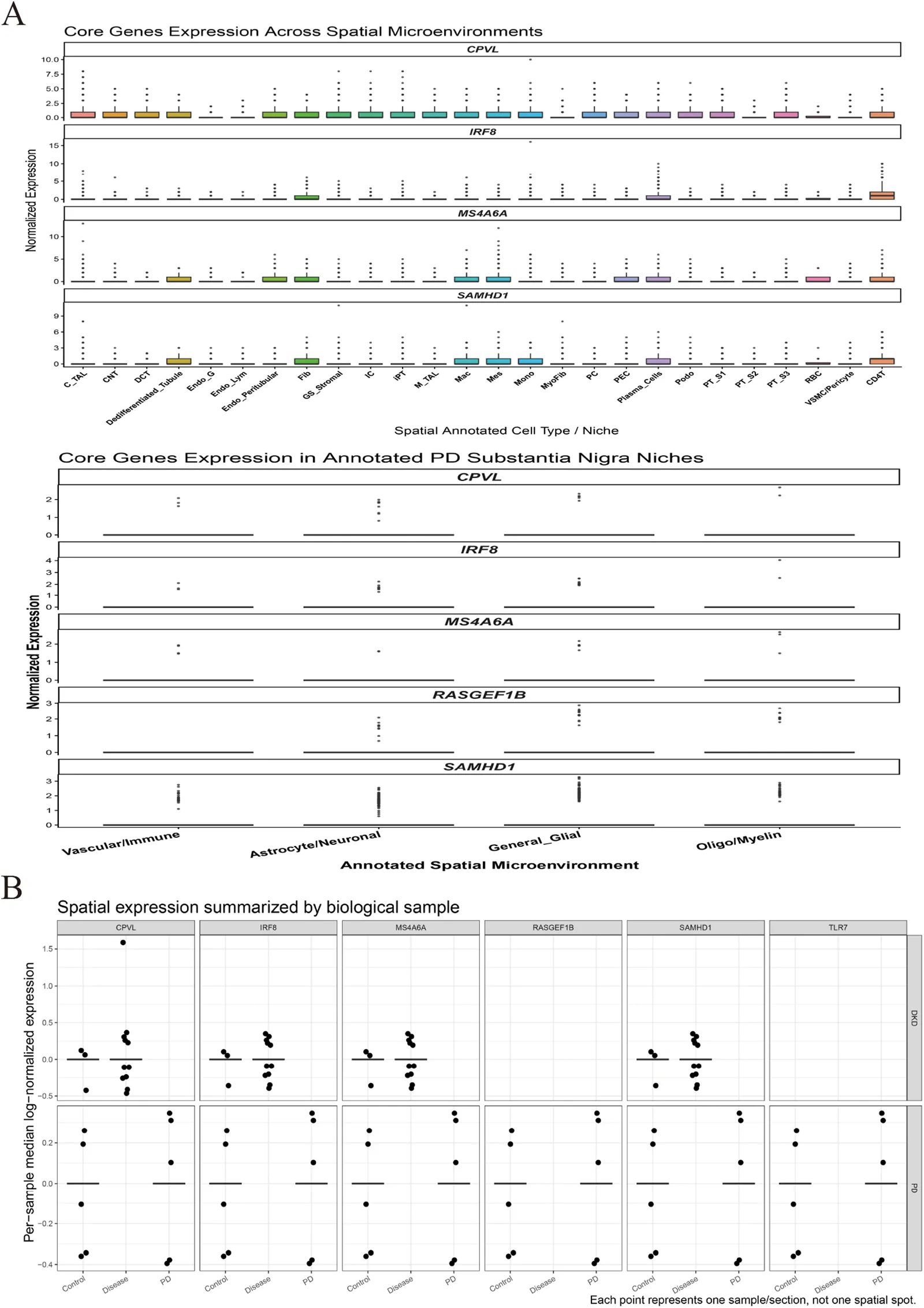
Marker-supported spatial localization and sample-level spatial statistics. (A) Spatial expression of IRF8/TLR7-associated candidate genes in DKD and PD tissues. The DKD Mac/Mono annotation was inherited from processed metadata and evaluated using macrophage/monocyte markers. The PD Vascular/Immune label represents a marker-supported interpretation of an unsupervised spatial cluster. (B) Sample- or section-level expression summaries. Each point represents one biological sample or spatial section rather than one individual spatial observation. Statistical comparisons were conducted using sample-level values to reduce pseudoreplication.

Spatial visualization showed localization of IRF8, TLR7, MS4A6A, SAMHD1, and CPVL within immune-associated regions of the DKD and PD spatial datasets (Fig 5A). The DKD Mac/Mono label was inherited from processed GSE211785 metadata and was supported by macrophage and monocyte markers. The PD Vascular/Immune label represented a marker-supported interpretation of an unsupervised cluster with combined vascular and immune expression and was not considered a histologically validated compartment. The lineage-marker expression patterns supporting these descriptive PD spatial niche annotations are shown in S5 Fig.

The original spot-pooled comparison was replaced by a sample- or section-level analysis. In revised Fig 5B, each point represents one biological sample or spatial section rather than one individual spot. The reanalysis therefore avoids treating spatially correlated observations as independent biological replicates. Given differences in dataset structure and the limited replication available for some spatial comparisons, these findings are interpreted as exploratory localization evidence rather than definitive spatial validation (Fig 5B; S5A–S5F Tables).

**Fig 6:**
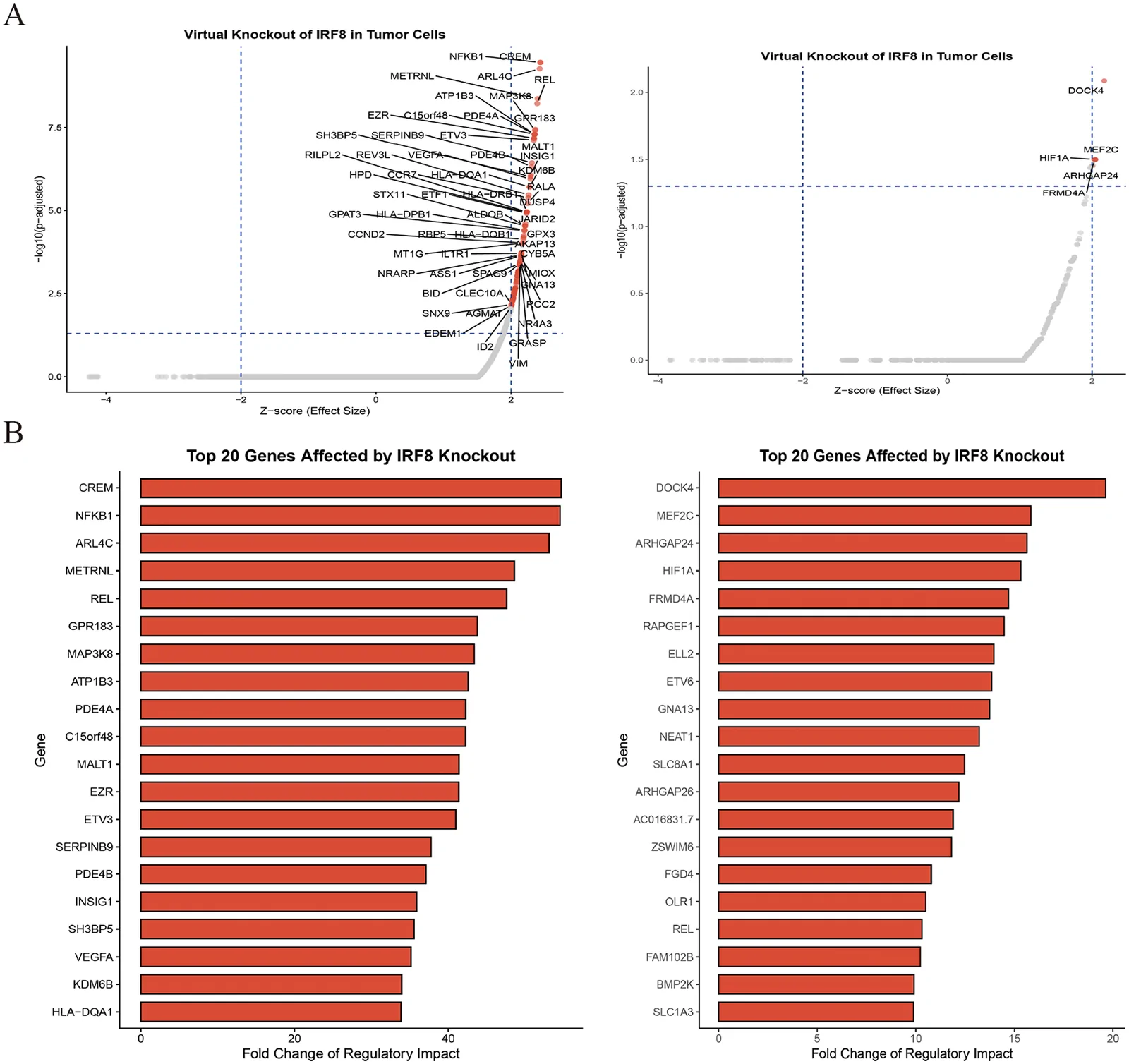
Predicted regulatory consequences of in-silico IRF8 deletion. (A) Volcano plots showing scTenifoldKnk-predicted differential regulation after computational removal of IRF8 outgoing network connections in DKD myeloid and PD microglial cells. (B) Top-ranked predicted regulatory changes. These results compare inferred baseline and pseudo-knockout networks and do not represent experimentally measured differential expression after IRF8 deletion.

The IRF8 perturbation analysis was performed using scTenifoldKnk rather than UNAGI or iDREM. Computational removal of the inferred outgoing IRF8 connections produced broad predicted differences between baseline and pseudo-knockout regulatory networks in DKD myeloid and PD microglial populations (Fig 6A). NFKB1, REL, HIF1A, and MEF2C were among the highly ranked predicted regulatory changes in one or both disease-specific analyses (Fig 6B).

These results nominate IRF8 as a candidate upstream regulator but do not demonstrate that IRF8 deletion causes the predicted transcriptomic changes. The disease-specific predictions were not validated using matched experimental knockout or perturbation datasets and are therefore presented as hypothesis-generating computational evidence.

### 3.6 High-resolution subclustering identifies candidate-enriched myeloid and microglial states

**Fig 7:**
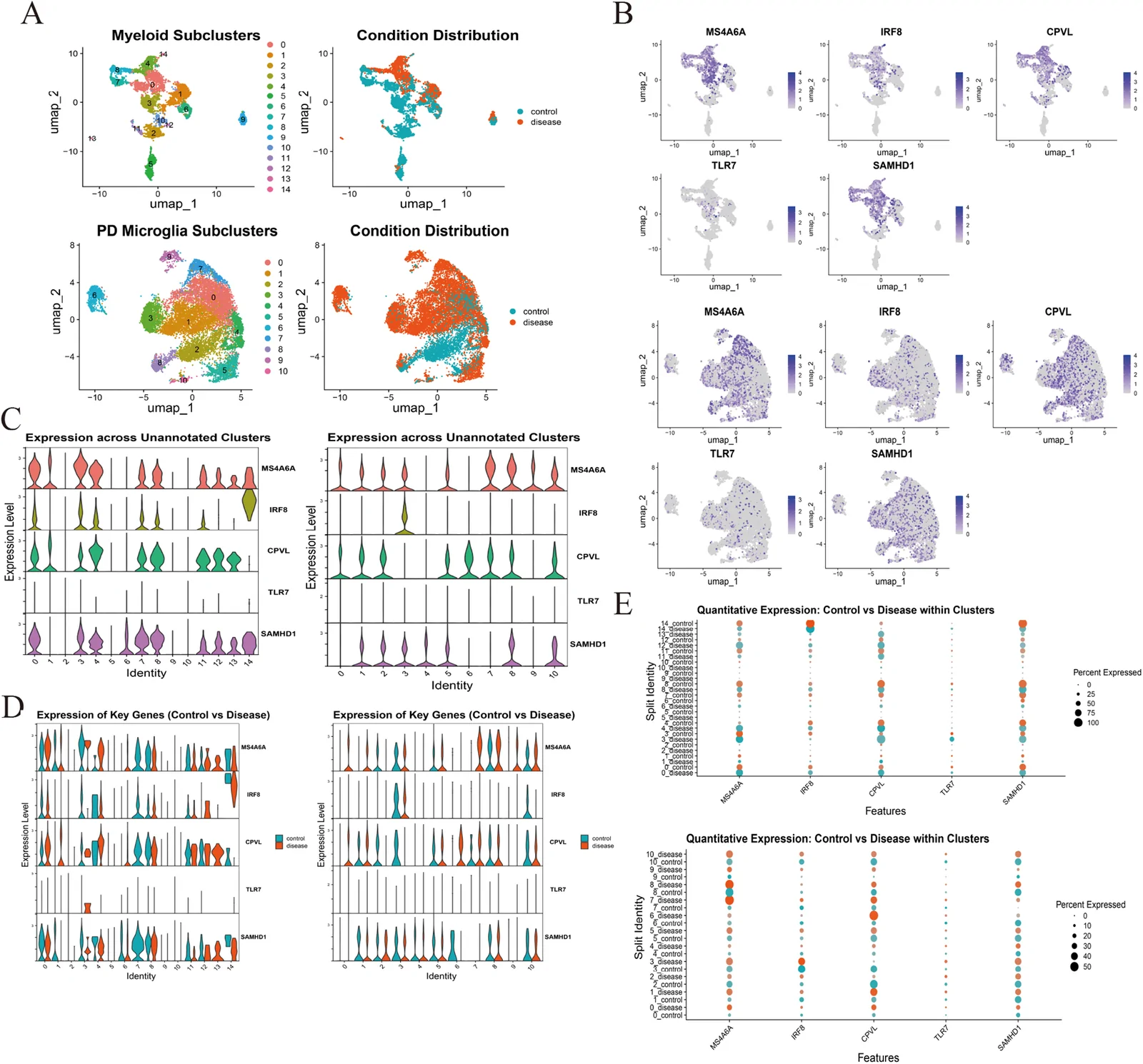
High-resolution subclustering of DKD myeloid and PD microglial/myeloid populations. (A) UMAP visualization of refined DKD and PD subclusters. (B) Feature plots showing the distribution of IRF8, TLR7, MS4A6A, SAMHD1, and CPVL. (C) Violin plots summarizing expression across subclusters. (D) Condition-split violin plots. (E) Condition-split dot plots. These visualizations identify candidate-enriched cellular states but do not establish cross-organ subcluster homology or therapeutic dependence.

High-resolution analysis identified 15 renal myeloid subclusters in DKD and 11 microglial/myeloid subclusters in PD (Fig 7A). IRF8, TLR7, MS4A6A, SAMHD1, and CPVL were not uniformly expressed across these populations. Instead, feature and violin plots showed enrichment within selected subclusters (Fig 7B-7C).

Condition-split violin and dot plots further indicated that the magnitude and distribution of candidate-gene expression differed between control and disease cells within several subclusters (Fig 7D-7E). These results identify cellular states in which the exploratory inflammatory candidates are most prominent. However, they do not by themselves establish that these subclusters are homologous across organs or that selectively targeting them would produce therapeutic benefit.

### 3.7 Normalized compound rankings prioritize NVP-AUY922 as a hypothesis-generating candidate

**Fig 8:**
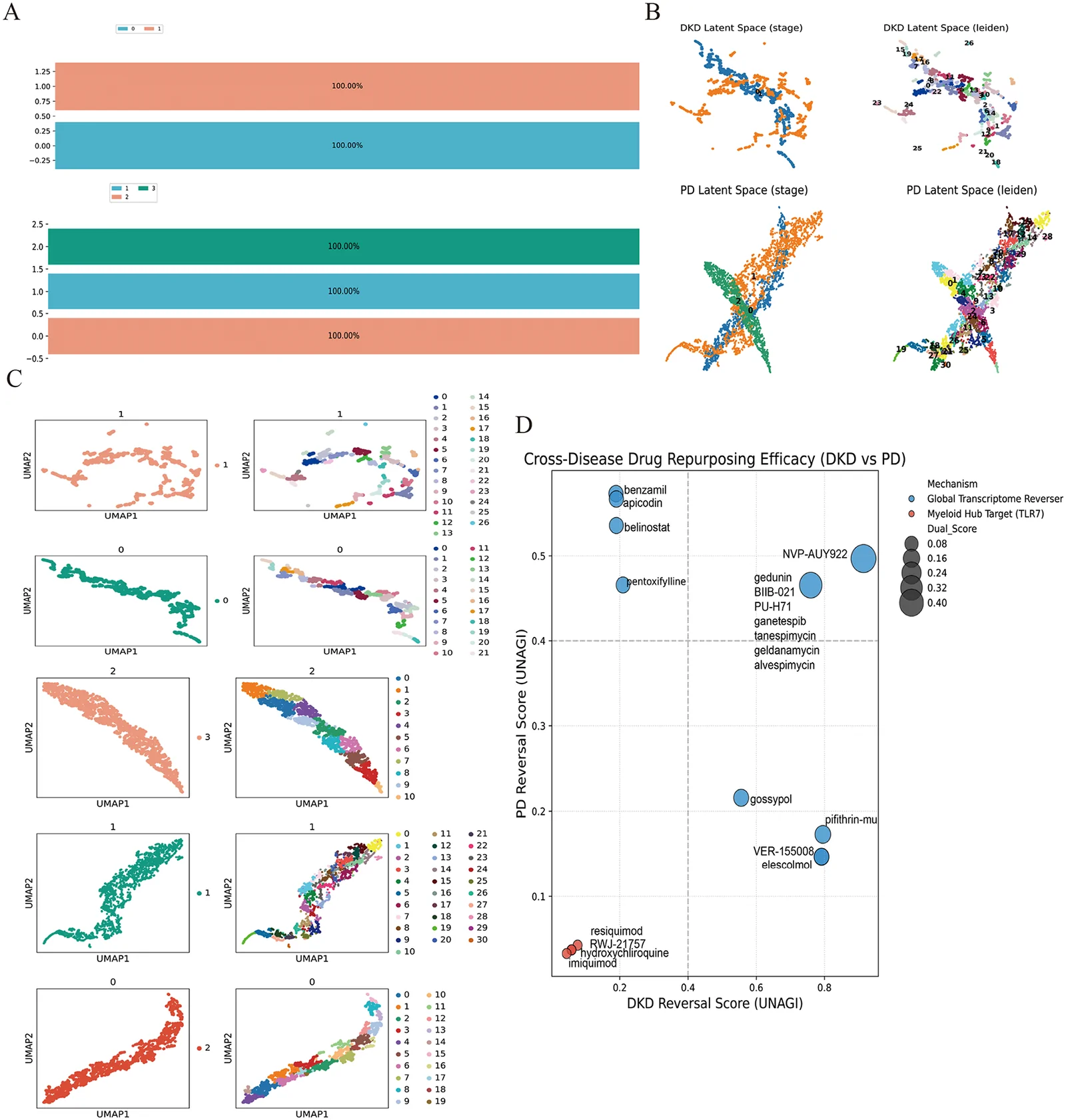
UNAGI latent representations and normalized cross-disease compound prioritization. (A) Cell-state composition across UNAGI latent groups. (B) Latent-space representations of DKD and PD cells. (C) Latent representations according to inferred disease-associated stage. (D) Cross-disease compound ranking based on the harmonic mean of within-disease percentile scores. NVP-AUY922 was the highest-ranked cross-disease computational candidate but did not remain statistically significant after correction across the complete compound universe.

UNAGI latent representations were used to summarize cellular-state structure and generate disease-specific compound rankings (Fig 8A–8C). Because the original DKD and PD compound scores were on potentially different scales, the scores were converted independently to within-disease percentiles before cross-disease comparison.

The harmonic-mean dual score prioritized compounds or compound groups that ranked consistently highly in both diseases (Fig 8D). Complete, top-30, and bottom-30 rankings are provided in S6A–S6D Tables. NVP-AUY922 ranked first in DKD, 26th in PD, and first in the normalized cross-disease analysis, with a dual-disease score of 0.9906. Its one-sided cross-disease empirical P value was 0.0205, but the result was not significant after correction across the complete matched compound universe (BH-FDR = 0.9527; S6E Table).

NVP-AUY922 is therefore described as the highest-ranked computational candidate rather than a statistically validated cross-disease treatment. The available perturbation resource did not retain cell-line, dose, treatment-duration, or individual perturbation identifiers, and the analysis did not establish myeloid- or microglia-specific pharmacological activity.

## 4. Discussion

This study evaluated cross-disease transcriptomic convergence between DKD and PD using independent microarray, single-cell or single-nucleus, spatial, communication, trajectory, regulatory-perturbation, and compound-prioritization analyses. The revised results support a more cautious conclusion than the original intersection-based analysis. DKD and PD do not show extensive, statistically robust genome-wide overlap under stringent meta-analytic criteria. Instead, the data identify a limited group of directionally concordant inflammatory candidates whose expression and predicted activity converge preferentially within renal myeloid cells and brain microglia.

The expanded bulk analysis is important for interpreting the scope of this convergence. The original 36-gene intersection depended on nominal thresholds and was not retained after cohort-wise BH correction. Moreover, none of the 12 originally prioritized upregulated genes passed genome-wide FDR correction in the PD and combined cross-disease analyses. IRF8 and TLR7 retained positive effects across diseases and sensitivity analyses, but these signals remained exploratory. Accordingly, the present study does not establish an IRF8/TLR7-driven shared pathogenic axis. Rather, it identifies IRF8/TLR7-associated transcriptional patterns that warrant evaluation in cell-type-specific and experimental systems [25,26].

The donor-level cell-composition analysis also changes the interpretation of the single-cell findings. No statistically significant increase in broad myeloid-lineage proportions was detected in either disease, but the wide confidence intervals—particularly in DKD, with only three donors per group—preclude a conclusion of equivalence [27]. The data therefore cannot distinguish completely between altered cell abundance and altered transcriptional state. Nevertheless, the preferential localization of IRF8, TLR7, MS4A6A, SAMHD1, and CPVL within renal myeloid and brain microglial populations indicates that the directionally concordant bulk signals are not uniformly distributed across all cell types [28].

The CellChat results further demonstrate that pathway-level labels should not be interpreted as uniformly activated across both disorders [21]. DKD-associated CCL interactions directed toward endothelial cells and several TNF interactions were reproducible across two balanced downsampling replicates. By contrast, the PD TGF-β result was retained in only one replicate, and other disease–pathway combinations were not reproducibly detected. These findings support specific hypotheses concerning endothelial–immune and lymphoid–immune communication in DKD but do not justify a general claim that TGF-β, TNF, and CCL signaling are equivalently enhanced in DKD and PD.

Monocle2 identified branched state spaces in both diseases, with non-random but overlapping distributions of control and disease cells. The moderate inverse correlations between primary- and reverse-root pseudotime indicate that the broad ordering was partly preserved when the trajectory was reoriented, but also confirm that pseudotime direction depends on root-state selection. In addition, pseudotime showed correlations with cellular quality metrics. The trajectories are therefore best interpreted as descriptive transcriptional-state continua rather than direct temporal records of disease progression. The revised analysis does not support the previous description of perfectly mirrored molecular evolution.

The spatial analyses provide anatomical context but have important limitations. The DKD Mac/Mono label was inherited from processed metadata, whereas the PD Vascular/Immune label was assigned through marker-supported interpretation of an unsupervised cluster. Neither annotation was generated through a new reference-based deconvolution procedure. Sample- and section-level reanalysis reduced the risk of pseudoreplication, but variation in spatial technologies, annotation strategies, and biological replication limits direct cross-organ comparison [29]. The spatial results therefore support localization of candidate expression within immune-associated regions but do not independently validate a shared causal mechanism.

The scTenifoldKnk analysis predicted broad regulatory-network changes after computational removal of IRF8 outgoing connections. This result provides a testable hypothesis that IRF8 may influence inflammatory regulatory structure in myeloid and microglial cells. However, it does not demonstrate that experimental IRF8 deletion would reproduce these changes. The baseline networks were inferred from observational control-cell expression data, and no matched CRISPR, knockout, or Perturb-seq dataset was available for disease-specific benchmarking. IRF8 should consequently be considered a candidate upstream regulator rather than a validated master regulator.

The normalized compound analysis prioritized NVP-AUY922, but the cross-disease association did not remain significant after multiple-testing correction. HSP90 inhibition has biological plausibility because HSP90 supports several stress- and inflammation-associated signaling proteins [30,31]. Nevertheless, HSP90 inhibitors may exert broad effects on stressed and proliferating cells, and the current analysis cannot distinguish selective reversal of a myeloid inflammatory state from a general cellular stress response. Blood–brain barrier exposure, systemic toxicity, renal safety, dosing, and activity in primary renal myeloid cells or human microglia require direct investigation. Similar caution applies to hydroxychloroquine and other TLR-associated candidates [32,33]. The present rankings should be used to prioritize experimental testing, not to recommend clinical use.

Several additional limitations should be considered. The analysis relied on heterogeneous public cohorts with incomplete clinical covariates, and uniform adjustment for disease stage, post-mortem interval, RNA quality, medication exposure, and comorbidities was not possible. Kidney and brain datasets were obtained from different participants and cannot directly establish a molecular mechanism underlying individual-level DKD–PD comorbidity. Single-cell differential patterns were largely evaluated through cell-level visualization, and future work should include adequately powered donor-level pseudobulk analyses. CellChat infers communication from expression of ligands and receptors and does not measure signaling activity directly. Pseudotime, spatial annotation, virtual perturbation, and compound prioritization are all model-dependent computational procedures requiring orthogonal validation.

## 5. Conclusion

The revised analyses identify partial cross-disease convergence of inflammatory myeloid and microglial transcriptional states associated with IRF8/TLR7-related signaling. The evidence supports a hypothesis-generating framework connecting kidney and brain innate immune responses but does not establish a conserved causal axis or validated cross-disease therapy. Prospective human cohorts, donor-level transcriptomic validation, experimental IRF8 perturbation, and pharmacological testing will be necessary to determine whether the prioritized programs and compounds have mechanistic or therapeutic relevance.

## Supporting information

S1 Fig Sensitivity and stability analyses of the bulk transcriptomic results. (A) Sensitivity to DEG thresholds. (B) Leave-one-DKD-cohort-out analysis. (C) Cross-disease meta-effect threshold sensitivity.
(TIF)

S2 Fig Sensitivity of donor-level cell-composition results to minimum retained-cell thresholds. Donor-level effect estimates were recalculated after applying minimum thresholds of 100, 500, and 1,000 retained cells per donor.
(TIF)

S3 Fig Monocle2 ordering-gene and root-orientation diagnostics. (A–B) Ordering-gene diagnostics for DKD and PD. (C–D) Relationships between primary- and reverse-root pseudotime values. (E–F) Reverse-root trajectory sensitivity analyses. Reverse-root analyses were used to assess trajectory orientation and were not interpreted as independent disease-progression models.
(TIF)

S4 Fig Targeted balanced-downsampling sensitivity analysis of CellChat results. Recurrence of TGF-β, TNF, and CCL pathway and ligand–receptor interactions across two independent balanced cell-number resampling replicates. Completed runs with no significant target-pathway interactions were included as zero-interaction results.
(TIF)

S5 Fig Marker support for PD spatial niche annotations. Expression of lineage-associated markers across PD spatial clusters used to support descriptive annotation of dopaminergic, microglial/myeloid, astrocytic, oligodendrocytic, lymphoid, vascular, and combined Vascular/Immune expression patterns. (Niche 0–4) in the Parkinson’s Disease (PD) midbrain sections.
(TIF)

S1 Table Supplementary dataset including study cohort and analytical outputs. (ZIP)

S2 Table Donor-level raw data, descriptive statistics, effect sizes and sensitivity analysis results. (ZIP)

S3 Table Trajectory analysis datasets and technical diagnostic information. (ZIP)

S4 Table Ligand–receptor interaction profiles and replicate recurrence analysis results. (ZIP)

S5 Table Dataset architecture, quality control records and statistical unit verification information. (ZIP)

S6 Table Complete feature rankings, top-30 and bottom-30 sorted results. (ZIP)
